# Supplementary material for: Setting Priorities to Inform Assessment of Care Homes’ Readiness to Participate in Healthcare Innovation: A Systematic Mapping Review and Consensus Process
Source: Int J Environ Res Public Health. 2020 Feb 5;17(3):987. doi: 10.3390/ijerph17030987 (PMC7037621; doi:10.3390/ijerph17030987)
Supplement: Supplementary file 1 [file ijerph-17-00987-s001.zip › Supp File 1.docx]

**Supplementary Table 1: Table of included studies**

| **Study ID** | **Study Type** | **Type of intervention** | | | | |  | **Categories of ACT** | | | | | | | |
| --- | --- | --- | --- | --- | --- | --- | --- | --- | --- | --- | --- | --- | --- | --- | --- |
|  |  | **Integrated working** | **Assessment** | **Telehealth** | **Other** | **Details if other** | **Related studies** | **L** | **C** | **E** | **SC** | **II** | **FI** | **SE** | **OS** |
| Aasmul 2018 [1] | PE |  | ✓ |  |  |  | Husebo 2015 [2] | ✓ | ✓ |  | ✓ | ✓ | ✓ | ✓ | ✓ |
| Agar 2017 [3] | RCT | ✓ |  |  |  |  | Lucket [4] | ✓ |  |  |  |  |  |  |  |
| Anderson 2011 [5] | RCT |  |  |  | ✓ | Multisensory therapeutic activity |  | ✓ | ✓ |  | ✓ | ✓ |  |  | ✓ |
| Arendts 2018 [6] | RCT | ✓ |  |  |  |  |  | ✓ | ✓ |  |  |  |  |  | ✓ |
| Ballard 2017 [7] | RCT | ✓ | ✓ |  |  |  | Fossey 2018, 2019 | ✓ |  | ✓ |  |  |  | ✓ |  |
| Beeckman 2013 | RCT |  |  |  | ✓ | Electronic clinical support system |  | ✓ | ✓ |  |  |  | ✓ | ✓ | ✓ |
| Beer 2011 [8] | RCT | ✓ |  |  |  |  | Beer 2011 [9] | ✓ | ✓ |  |  |  | ✓ |  | ✓ |
| Beer 2011 [9] | PE | ✓ |  |  |  |  | Beer 2011 [8] | ✓ | ✓ |  |  |  |  |  | ✓ |
| Bleckken 2015 [10] | RCT |  |  |  | ✓ | Educational programme for faecal incontinence |  | ✓ | ✓ |  |  |  |  | ✓ | ✓ |
| Boorsma 2011 [11] | RCT | ✓ |  |  |  |  |  | ✓ |  |  | ✓ |  |  | ✓ |  |
| Boyd 2014 [12] | RCT | ✓ |  |  |  |  |  | ✓ |  |  |  |  |  |  | ✓ |
| Brazil 2018 [13] | RCT |  | ✓ |  |  |  |  |  |  | ✓ |  |  |  | ✓ | ✓ |
| Brodaty 2014 [14] | RCT |  |  |  | ✓ | Laughter therapy |  | ✓ |  |  |  |  |  |  | ✓ |
| Brooker 2016 [15] | RCT |  |  |  | ✓ | Person centred care |  | ✓ | ✓ |  |  | ✓ | ✓ | ✓ | ✓ |
| Chami 2012 [16] | RCT |  |  |  | ✓ | Infection prevention |  | ✓ |  |  |  |  |  |  | ✓ |
| Chi 2010 [17] | RCT | ✓ | ✓ |  |  |  |  |  |  |  |  | ✓ |  | ✓ | ✓ |

| **Study ID** | **Study Type** | **Type of intervention** | | | | |  | **Categories of ACT (considered pre or post hoc)** | | | | | | | |
| --- | --- | --- | --- | --- | --- | --- | --- | --- | --- | --- | --- | --- | --- | --- | --- |
|  |  | **Integrated working** | **Assessment** | **Telehealth** | **Other** | **Details if other** | **Related studies** | **L** | **C** | **E** | **SC** | **II** | **FI** | **SE** | **OS** |
| Close 2013 [18] | Qual | ✓ |  |  |  |  | Hancock (not included) | ✓ |  |  |  | ✓ |  |  |  |
| Cohen-Mansfield 2012 [19] | PE |  |  |  | ✓ | Non pharmacological intervention for behavioural symptoms |  |  |  |  |  |  | ✓ | ✓ | ✓ |
| Colon_Emeric 2014 [20] | PE |  |  |  | ✓ | Fall prevention | Colon Emeric 2014 [21] | ✓ | ✓ |  |  |  |  |  | ✓ |
| Colon-Emeric 2014 [21] | RCT |  |  |  | ✓ | Fall prevention | Colon Emeric 2014 [20] | ✓ |  |  |  | ✓ |  |  | ✓ |
| Connolly 2015 [22] | RCT | ✓ |  |  |  |  |  |  | ✓ |  |  |  | ✓ |  | ✓ |
| Crotty 2004 [23] | RCT | ✓ |  |  |  |  |  | ✓ |  |  |  |  | ✓ |  | ✓ |
| Davison 2013 [24] | RCT |  |  |  | ✓ | Education to promote screening for depression |  | ✓ |  |  |  |  | ✓ |  | ✓ |
| De Visschere 2012 [25] | RCT |  |  |  | ✓ |  |  | ✓ |  |  |  | ✓ | ✓ | ✓ | ✓ |
| Dorsey 2010 [26] | RCT |  |  | ✓ |  |  |  | ✓ | ✓ |  | ✓ |  | ✓ | ✓ |  |
| Dozeman 2012 [27] | RCT | ✓ |  |  |  |  |  |  |  |  |  |  |  |  |  |
| Ellard 2014 [28] | PE |  |  |  | ✓ | Physical activity for depression | Underwood [29] | ✓ | ✓ |  | ✓ |  | ✓ |  | ✓ |
| Field 2011 [30] | RCT |  |  | ✓ |  |  |  |  |  |  |  |  |  |  | ✓ |
| Fossey 2018 [31] | Qual | ✓ | ✓ |  |  |  | Ballard [7] | ✓ | ✓ |  | ✓ |  | ✓ | ✓ | ✓ |
| Fossey 2018 [32] | Qual | ✓ | ✓ |  |  |  | Ballard [7] | ✓ | ✓ | ✓ | ✓ | ✓ | ✓ | ✓ | ✓ |

| **Study ID** | **Study Type** | **Type of intervention** | | | | |  | **Categories of ACT** | | | | | | | |
| --- | --- | --- | --- | --- | --- | --- | --- | --- | --- | --- | --- | --- | --- | --- | --- |
|  |  | **Integrated working** | **Assessment** | **Telehealth** | **Other** | **Details if other** | **Related studies** | **L** | **C** | **E** | **SC** | **II** | **FI** | **SE** | **OS** |
| Fossey 2019 [33] | Qual | ✓ | ✓ |  |  |  | Ballard [7] | ✓ | ✓ | ✓ | ✓ | ✓ | ✓ | ✓ | ✓ |
| Gage 2012 [34] | Survey | ✓ |  |  |  |  |  | ✓ | ✓ |  | ✓ |  | ✓ |  |  |
| Greenspan 2012 [35] | PE |  |  |  | ✓ | Calcium for osteoporosis |  |  | ✓ |  | ✓ |  | ✓ |  | ✓ |
| Hall 2009 [36] | Qual |  |  |  | ✓ | Challenges conducting research |  |  |  |  |  |  |  |  | ✓ |
| Husebo 2019 [2] | RCT | ✓ | ✓ |  |  |  |  | ✓ |  | ✓ |  |  | ✓ | ✓ | ✓ |
| Innis 2016 [37] | Literature review |  |  |  | ✓ | Study of nurse managers |  | ✓ | ✓ |  | ✓ | ✓ | ✓ | ✓ | ✓ |
| Kinley 2014 [38] | RCT |  |  |  | ✓ | Facilitation for implementation of gold standard framework |  | ✓ | ✓ |  |  |  | ✓ |  | ✓ |
| Livingston 2019 [39] | RCT | ✓ |  |  |  |  |  | ✓ | ✓ | ✓ |  |  | ✓ | ✓ | ✓ |
| Lucket 2017 | Qual | ✓ |  |  |  |  | Agar | ✓ | ✓ |  |  |  | ✓ |  | ✓ |
| McSweeney 2012 | RCT | ✓ |  |  |  |  |  |  |  |  |  |  |  |  |  |
| Meeks 2015 | RCT |  |  |  | ✓ | Behavioural intervention for depression |  |  | ✓ |  |  |  |  |  | ✓ |
| Overbeek 2018 | RCT |  | ✓ |  |  |  |  | ✓ | ✓ | ✓ |  |  | ✓ | ✓ |  |
| Oye 2016 | Qual |  |  |  | ✓ | Assess how contextual factors influenced implementation |  | ✓ | ✓ |  |  |  | ✓ |  | ✓ |
| Palmer 2018 [40] | RCT |  | ✓ |  |  |  |  | ✓ | ✓ | ✓ |  |  | ✓ | ✓ | ✓ |
| Poot 2016 | PE | ✓ |  |  |  |  |  |  |  |  |  |  | ✓ |  |  |

| **Study ID** | **Study Type** | **Type of intervention** | | | | |  | **Categories of ACT** | | | | | | | |
| --- | --- | --- | --- | --- | --- | --- | --- | --- | --- | --- | --- | --- | --- | --- | --- |
|  |  | **Integrated working** | **Assessment** | **Telehealth** | **Other** | **Details if other** | **Related studies** | **L** | **C** | **E** | **SC** | **II** | **FI** | **SE** | **OS** |
| Rantz 2012 | RCT | ✓ |  |  |  |  |  | ✓ |  |  |  |  | ✓ |  | ✓ |
| Shepherd 2015 | PE |  |  |  | ✓ | Challenges setting up an RCT |  |  | ✓ |  | ✓ |  | ✓ |  | ✓ |
| Simpson 2013 | PE |  |  |  | ✓ | Development of tool for addressing implementation challenges | Colon-emeric [20, 41] | ✓ | ✓ |  |  |  |  |  | ✓ |
| Stein-Parbury 2012 | PE |  |  |  | ✓ |  | Chenoweth 2009 (not included) | ✓ | ✓ |  |  |  |  |  |  |
| Stern 2014 | RCT | ✓ |  |  |  |  |  | ✓ | ✓ |  |  |  |  |  | ✓ |
| Surr 2019 [42] | RCT |  |  |  | ✓ | Dementia care mapping |  | ✓ | ✓ | ✓ | ✓ | ✓ | ✓ | ✓ | ✓ |
| Underwood 2013 | RCT | ✓ |  |  |  |  |  |  |  | ✓ |  |  |  |  | ✓ |
| van der Kooij 2013 | RCT |  |  |  | ✓ | Integrated emotion orientated care |  | ✓ |  | ✓ |  |  |  |  |  |
| Van Ness 2012 | PE |  |  |  | ✓ | Methodological issues of conducting trials in care homes |  | ✓ | ✓ |  |  |  |  |  | ✓ |
| Vowden 2013 | RCT |  |  | ✓ |  |  |  |  |  |  |  |  |  | ✓ |  |
| Wenborn 2013 | RCT | ✓ |  |  |  |  |  | ✓ | ✓ |  |  |  | ✓ | ✓ | ✓ |

RCT = randomised controlled trial, PE = process evaluation

Categories of ACT: L = leadership, C = culture, E = evaluation, SC = social capital, II = informal interactions, FI = formal interactions, SE = structural electronic resources, OS = organisational slack

References

1. Aasmul I, Husebo BS, Flo E. Description of an advance care planning intervention in nursing homes: outcomes of the process evaluation. BMC Geriatr. 2018;18:26.

2. Husebo BS, Ballard C, Aarsland D, Selbaek G, Slettebo DD, Gulla C, et al. The Effect of a Multicomponent Intervention on Quality of Life in Residents of Nursing Homes: A Randomized Controlled Trial (COSMOS). J Am Med Dir Assoc. 2019;20:330–9.

3. Agar M, Luckett T, Luscombe G, Phillips J, Beattie E, Pond D, et al. Effects of facilitated family case conferencing for advanced dementia: A cluster randomised clinical trial. PLoS One. 2017;12:e0181020.

4. Luckett T, Chenoweth L, Phillips J, Brooks D, Cook J, Mitchell G, et al. A facilitated approach to family case conferencing for people with advanced dementia living in nursing homes: perceptions of palliative care planning coordinators and other health professionals in the IDEAL study. Int Psychogeriatrics. 2017;29:1713–22. doi:DOI: 10.1017/S1041610217000977.

5. Anderson K, Bird M, Macpherson S, McDonough V, Davis T. Findings from a pilot investigation of the effectiveness of a snoezelen room in residential care: should we be engaging with our residents more? Geriatr Nurs. 2011;32:166–77.

6. Arendts G, Deans P, Brien KO, Etherton-beer C, Lewin G, Sim M, et al. A clinical trial of nurse practitioner care in residential aged care facilities. Arch Gerontol Geriatr. 2018;77 May:129–32. doi:10.1016/j.archger.2018.05.001.

7. Ballard C, Orrell M, Sun Y, Moniz-Cook E, Stafford J, Whitaker R, et al. Impact of antipsychotic review and non-pharmacological intervention on health-related quality of life in people with dementia living in care homes: WHELD—a factorial cluster randomised controlled trial. Int J Geriatr Psychiatry. 2017;32:1094–103.

8. Beer C, Horner B, Flicker L, Scherer S, Lautenschlager NT, Bretland N, et al. A cluster-randomised trial of staff education to improve the quality of life of people with dementia living in residential care: the DIRECT study. PLoS One. 2011;6:e28155.

9. Beer C, Lowry R, Horner B, Almeida OP, Scherer S, Lautenschlager NT, et al. Development and evaluation of an educational intervention for general practitioners and staff caring for people with dementia living in residential facilities. Int Psychogeriatr. 2011;23:221–9.

10. Blekken LE, Nakrem S, Gjeilo KH, Norton C, Morkved S, Vinsnes AG. Feasibility, acceptability, and adherence of two educational programs for care staff concerning nursing home patients’ fecal incontinence: a pilot study preceding a cluster-randomized controlled trial. Implement Sci. 2015;10:72.

11. Boorsma M, Frijters DH, Knol DL, Ribbe ME, Nijpels G, van Hout HP. Effects of multidisciplinary integrated care on quality of care in residential care facilities for elderly people: a cluster randomized trial. Cmaj. 2011;183:E724-32.

12. Boyd M, Armstrong D, Parker J, Pilcher C, Zhou L, McKenzie-Green B, et al. Do gerontology nurse specialists make a difference in hospitalization of long-term care residents? Results of a randomized comparison trial. J Am Geriatr Soc. 2014;62:1962–7.

13. Brazil K, Carter G, Cardwell C, Clarke M, Hudson P, Froggatt K, et al. Effectiveness of advance care planning with family carers in dementia nursing homes: A paired cluster randomized controlled trial. Palliat Med. 2018;32:603–12.

14. Brodaty H, Low LF, Liu Z, Fletcher J, Roast J, Goodenough B, et al. Successful ingredients in the SMILE study: Resident, staff, and management factors influence the effects of humor therapy in residential aged care. Am J Geriatr Psychiatry. 2014;22:1427–37. doi:10.1016/j.jagp.2013.08.005.

15. Brooker DJ, Latham I, Evans SC, Jacobson N, Perry W, Bray J, et al. FITS into practice: translating research into practice in reducing the use of anti-psychotic medication for people with dementia living in care homes. Aging Ment Health. 2015;7863 March 2016:1–10. doi:10.1080/13607863.2015.1063102.

16. Chami K, Gavazzi G, Bar-Hen A, Carrat F, de Wazières B, Lejeune B, et al. A Short-Term, Multicomponent Infection Control Program in Nursing Homes: A Cluster Randomized Controlled Trial. J Am Med Dir Assoc. 2012;13:569.e9-569.e17 1p. doi:10.1016/j.jamda.2012.04.008.

17. Chi I, Law B V, Leung AC, Liu CP, Yeoh CS, Cheng YH, et al. Residential Assessment Instrument 2.0 in care planning for residents in nursing homes. Hong Kong Med J. 2010;16 Suppl 3:29–33.

18. Close H, Hancock H, Mason JM, Murphy JJ, Fuat A, de Belder M, et al. “It’s Somebody else’s responsibility” - perceptions of general practitioners, heart failure nurses, care home staff, and residents towards heart failure diagnosis and management for older people in long-term care: a qualitative interview study. BMC Geriatr. 2013;13:69.

19. Cohen-mansfield. What are the barriers to performing nonpharmalogical interventions for Behavioral symptoms in the nursing home? J Am Med Dir Assoc. 2012;100:130–4.

20. Colon-Emeric CS, Pinheiro SO, Anderson RA, Porter K, McConnell E, Corazzini K, et al. Connecting the learners: improving uptake of a nursing home educational program by focusing on staff interactions. Gerontologist. 2014;54:446–59.

21. Colon-Emeric CS, McConnell E, Pinheiro SO, Corazzini K, Porter K, Earp KM, et al. CONNECT for better fall prevention in nursing homes: results from a pilot intervention study. J Am Geriatr Soc. 2013;61:2150–9.

22. Connolly MJ, Boyd M, Broad JB, Kerse N, Lumley T, Whitehead N, et al. The Aged Residential Care Healthcare Utilization Study (ARCHUS): A Multidisciplinary, Cluster Randomized Controlled Trial Designed to Reduce Acute Avoidable Hospitalizations From Long-Term Care Facilities. J Am Med Dir Assoc. 2015;16:49–55.

23. Crotty M, Halbert J, Rowett D, Giles L, Birks R, Williams H, et al. An outreach geriatric medication advisory service in residential aged care: a randomised controlled trial of case conferencing. Age Ageing. 2004;33:612-617 6p. http://search.ebscohost.com/login.aspx?direct=true&db=jlh&AN=106627237&site=ehost-live.

24. Davison TE, Karantzas G, Mellor D, McCabe MP, Mrkic D. Staff-focused interventions to increase referrals for depression in aged care facilities: a cluster randomized controlled trial. Aging Ment Heal. 2013;17:449–55.

25. De Visschere L, Schols J, van der Putten GJ, de Baat C, Vanobbergen J. Effect evaluation of a supervised versus non-supervised implementation of an oral health care guideline in nursing homes: a cluster randomised controlled clinical trial. Gerodontology. 2012;29:e96-106.

26. Dorsey ER, Deuel LM, Voss TS, Finnigan K, George BP, Eason S, et al. Increasing access to specialty care: a pilot, randomized controlled trial of telemedicine for Parkinson’s disease. Mov Disord. 2010;25:1652–9.

27. Dozeman E, Marwijk HWJ Van, Schaik DJF Van, Smit F, Stek ML, Horst E Van Der, et al. Contradictory effects for prevention of depression and anxiety in residents in homes for the elderly : a pragmatic randomized controlled trial. Int Pscychogeriatrics. 2012;24:1242–51.

28. Ellard DR, Thorogood M, Underwood M, Seale C, Taylor SJ. Whole home exercise intervention for depression in older care home residents (the OPERA study): a process evaluation. BMC Med. 2014;12:1.

29. Underwood M, Lamb SE, Eldridge S, Sheehan B, Slowther AM, Spencer A, et al. Exercise for depression in elderly residents of care homes: a cluster-randomised controlled trial. Lancet. 2013;382:41–9.

30. Field TS, Tjia J, Mazor KM, Donovan JL, Kanaan AO, Harrold LR, et al. Randomized trial of a warfarin communication protocol for nursing homes: an SBAR-based approach. Am J Med. 2011;124:179.e1-7.

31. Fossey J, Garrod L, Guzman A, Testad I. A qualitative analysis of trainer/coach experiences of changing care home practice in the Well-being and Health in Dementia randomised control trial. Dementia. 2018.

32. Fossey J, Garrod L, Lawrence V, Testad I, Stafford J, Murray J. “We should see her like part of the team”: an investigation into care home staff’s experiences of being part of an RCT of a complex psychosocial intervention. Aging Ment Heal. 2018;0:1–8. doi:10.1080/13607863.2018.1525603.

33. Fossey J, Garrod L, Tolbol Froiland C, Ballard C, Lawrence V, Testad I. What influences the sustainability of an effective psychosocial intervention for people with dementia living in care homes? A 9 to 12-month follow-up of the perceptions of staff in care homes involved in the WHELD randomised controlled trail. Int J Geriatr Psychiatry. 2019;34:674–82.

34. Gage H, Dickinson A, Victor C, Williams P, Cheynel J, Davies SL, et al. Integrated working between residential care homes and primary care: a survey of care homes in England. BMC Geriatr. 2012;12:71.

35. Greenspan SL, Nace MD, Perera S FM et al. Lessons Learned from an Osteoporosis Clinical Trial in Frail Long Term Care Residents. Clin Trials. 2012;100:130–4.

36. Hall S, Longhurst S, Higginson IJ. Challenges to conducting research with older people living in nursing homes. BMC Geriatr. 2009;9:38.

37. Innis J, Berta W. Routines for change: how managers can use absorptive capacity to adopt and implement evidence-based practice. J Nurs Manag. 2016;:n/a-n/a. doi:10.1111/jonm.12368.

38. Kinley, Stone L, Dewey M, Levy J, Stewart R, McCrone P, et al. The effect of using high facilitation when implementing the Gold Standards Framework in Care Homes programme: A cluster randomised controlled trial. Palliat Med. 2014;28:1099–109. doi:10.1177/0269216314539785.

39. Livingston G, Barber J, Marston L, Stringer A, Panca M, Hunter R, et al. Clinical and cost-effectiveness of the Managing Agitation and Raising Quality of Life (MARQUE) intervention for agitation in people with dementia in care homes: a single-blind, cluster-randomised controlled trial. The Lancet Psychiatry. 2019;6:293–304. doi:10.1016/S2215-0366(19)30045-8.

40. Palmer JA, Mor V, Volandes AE, McCreedy E, Loomer L, Carter P, et al. A dynamic application of PRECIS-2 to evaluate implementation in a pragmatic, cluster randomized clinical trial in two nursing home systems. Trials. 2018;19:453.

41. Colon-emeric CS, Mcconnell E, Sandro O, Corazzini K, Porter K, Earp KM, et al. CONNECT for Better Fall Prevention in Nursing Homes: Results from a Pilot Intervention Study. J Am Geriatr Soc. 2014;61:2150–9.

42. Surr CA, Holloway I, Walwyn REA, Griffiths AW, Meads D, Kelley R, et al. Dementia Care Mapping TM to reduce agitation in care home residents with dementia : The DCM ^TM^ EPIC cluster randomised controlled trial. Health Technol Assess (Rockv). 2019;:1–239.
