# Supplementary material for: Setting Priorities to Inform Assessment of Care Homes’ Readiness to Participate in Healthcare Innovation: A Systematic Mapping Review and Consensus Process
Source: Int J Environ Res Public Health. 2020 Feb 5;17(3):987. doi: 10.3390/ijerph17030987 (PMC7037621; doi:10.3390/ijerph17030987)
Supplement: Supplementary file 1 [file ijerph-17-00987-s001.zip › Supp File 2.docx]

**Supplementary Table 2: Summary of the ACT domains and linked evidence from the mapping review**

| **Domain: Leadership**  **Defined as: The actions of formal leaders in an organization to influence change and excellence in practice. Positive leadership linked to higher research use.** | |
| --- | --- |
| *Positive impact of context on implementation* | *Negative impact of context on implementation* |
| - Leadership of nurses and/or staff members identified as clinical *champions*’ or persuasive leaders [1] [2][3][4][5][6][7][8] - Evidence of nursing home administrator and/or director of nursing support for team efforts [9, 10] Managers present in the care home [11] - Both care manager and opinion leader [12] staff member to support at unit level of care home [13] - Engaged leadership and management support [14] [15][8][7] - National endorsement of importance of the intervention [16] - Targeting engagement of NH managers facilitated engagement [17, 18] | - High turnover of care-home managers [19] - Leadership under formal investigation [20] - Resistance or lack of interest from management [21][22, 23] - Increased leadership turnover increased turnover of direct-care workers [9] - Insufficient management attention [24][12] - Unclear who took responsibility for certain residents [25] - Few care homes identified a *champion* [26] - Lack of management support, especially when they didn’t have a clear understanding of the intervention [27] |
| **Domain: Culture**  **Defined as: The way that “we do things” in the organization; items indicative of a supportive work culture. Positive culture linked to higher research use.** | |
| *Positive impact of context on implementation* | *Negative impact of context on implementation* |
| - Consultation with stakeholders to discuss program development and implementation [20][8][28][29] - Evidence of  willingness to change and a culture of learning, collaboration, and quality improvement [30–32] - Staff had prior experience using telemedicine to provide Parkinson’s disease care [33] - Protocol fitted with existing ways of working [34, 35] - A positive care community essential for change [13] - Care planning and protocols and staff timetabling/regimens on interventions aligned [14] - Evidence of trusting working relationships with different organisations [36] - Fits with priorities of managers [5] - Attempted to integrate strategies for change into routine practice [6] | - Perception that the new model was denigrating existing care models [37] - Residents with complex needs/heterogeneity of residents [12][38][39][40] - Low staff morale [26] - Resistant to change to existing processes [1][41] - Research naïve environment [42] - Belief that there is no time for extra activities in homes [9] - negative attitudes to intervention [8, 43] - Staff focused on tasks [11, 15] - Units in care homes have different cultures [13][36] - Preoccupation with risk and protection from harm [25][26] - Staff prioritise residents’ privacy [44] - Difficulty of challenging long established practices [8] - Staff don’t see sitting and talking with residents as real work [27] |
| **Domain: Evaluation**  **Defined as: The process of using data to assess group/team performances & to achieve outcomes in organizations or units. Larger number of unit feedback mechanisms linked to higher research use.** | |
| *Positive impact of context on implementation* | *Negative impact of context on implementation* |
| - Systematic approach to resident assessment [19] - Identification of clinical topics for quality improvement that could be measured and evaluated [9] - Able to do on-site examinations and tests [35] - Change requires guidance over time, creates a sense of ownership with feedback data on staffing and resident characteristics [10] - Staff familiar with innovation approach [45] | - Managing and synthesising different types of data challenging [22, 26, 36] - Assessment and/or MDS data not used by staff [41, 46] |
| **Domain: Social Capital**  **Defined as: The stock of active connections among people. These connections are of three types: bonding, bridging, and linking. More positive social capital linked to higher research use.** | |
| *Positive impact of context on implementation* | *Negative impact of context on implementation* |
| - Physician present and working with multidisciplinary team [19] - Nurse specialist involved in overseeing residents’ care across organisations [20] - Academic support for introduction of intervention (telemedicine) [33] - Care homes excluded if they had already participated in project to avoid staff overload [10] - Staff paid to do the intervention [35] - Good relationships with the local community [8] - Development of trust with care home teams was a gradual process [8, 43] - Acknowledging the expertise of the care home staff acting as champions [8, 43] | - Similarity to another recent study in which care home had participated [47] - Already involved in other time-demanding projects [13] - Visiting physicians/GPs unreceptive to change, unwilling to engage [3, 12, 25, 30, 33] - Need for therapist involvement [11] |
| **Domain: Informal interactions**  **Defined as: Information exchanges that occur between individuals working within an organization (unit) that can promote the transfer of knowledge. Larger number of informal interactions linked to higher research use.** | |
| *Positive impact of context on implementation* | *Negative impact of context on implementation* |
| - Management ongoing support and discussions about an intervention affects staff commitment to that intervention, this affects residents’ response and outcomes [14] - Opportunities to build relationships with care home staff [22] - Ongoing involvement with the research therapist [8] - Research team available to discuss queries [8, 43, 48] | - Enthusiasm of leadership not communicated to staff [11] - Lack of opportunity to discuss risks and benefits [25] - Caregivers find it difficult to convince residents to follow the new guidelines, resistant to change - Staff unaware of intervention due to poor internal communication [26][21] |
| **Domain: Formal interactions**  **Defined as: Formal exchanges that occur between individuals working within an organization (unit) through scheduled activities that can promote the transfer of knowledge. Larger number of formal interactions linked to higher research use.** | |
| *Positive impact of context on implementation* | *Negative impact of context on implementation* |
| - Case conferences more frequent in the intervention group [1] - An advisory group, that included representatives, from health, staff, residents, and old age charity, advised before and during the study [47] - Information about meetings readily available, all levels of staff involved meetings, discussion about the clinical topics being addressed [9, 34] - Coaching to improve knowledge and skills to increase confidence of care givers [10][6, 8, 43, 48] - Regular contact between researchers and care home staff [17][27] | - Low GP participation/ lacked formal communication & Multidisciplinary team(MDT) planning and involvement of GP and or family affected engagement [1, 36, 41, 42, 49] - Disagreement between staff members about what should be done [25] - Attrition rates coincided with workload pressures exacerbated by poor communication regarding training requirements and travel distances [22] - Lack of trust in care home staff expertise [36] - Long process for approval to participate [42] |
| **Domain; Structural/Electronic Resources**  **Defined as: The structural and electronic elements of an organization (unit) that facilitate the ability to assess and use knowledge** | |
| *Positive impact of context on implementation* | *Negative impact of context on implementation* |
| - Specialist role possible within funding constraints [20] - Technology becoming easier to use and relatively inexpensive [33] - Active engagement in research process led to improvements in routine collection of data because staff could see the link between practice and outcomes [8, 43] - Research team provide support for IT issues [7] - Manualised intervention [6, 27] | - Funding system [19][40] - Funding model did not support/could not afford the intervention [49][2][50] - Need time for staff to iron out technical problems and become familiar with technology [51] - Limited availability of materials and the high cost may have limited uptake (Beeckman et al., 2013) - Difficult to share data [36] - Too few computers in care home [13][27] - Teleconference supervisions disliked and eventually rejected [22] - Need resources to support change [16] - Manual could initially be overwhelming [8] |
| **Domain: Organisational Slack staff**  **Defined as: The cushion of actual or potential resources which allows an organization (unit) to adapt successfully to internal pressures for adjustments or to external pressures for changes. Sufficient staffing levels linked to higher research use** | |
| *Positive impact of context on implementation* | *Negative impact of context on implementation* |
| - When staff had time to participate in the meetings and interventions this improved residents’ quality of life [1] - Specialist role possible with access to nurses with relevant expertise [20] - After staff believed in intervention/ saw improvements, changes became easier (consolidate gains produce more change) [9, 14] - Staff with the right skills were crucial to care home being able to implement wound care intervention [24] - Agency and temporary staff were not included in the innovation [2] - Clearly defined roles and responsibilities [17, 18] - Reimbursement for staff cover during training sessions [6] | - Staff turnover affected ability to learn new skills [37][24] - Low staff participation/beyond scope of role and responsibilities [1][26] - Could not employ nurse practitioners (NPs), who could prescribe medication [47] - Limited staff availability or capacity to take on new roles [22, 29, 51, 52][5][27][7] - Case conferences, group discussions, and formal interactions difficult to organise [49][32] - Could not incorporate into workload [12, 53] - Staff lacked confidence and needed extra training and support not available in the work setting [30] - No cover/funding for staff to attend meetings or participate [3, 9, 21, 40, 42] - Not a staff priority [11, 46] - Nursing role unclear [13] - Literacy levels of staff when using manuals [8] |
| **Domain: Organisational Slack- Space**  **Definition: The cushion of actual or potential resources which allows an organization (unit) to adapt successfully to internal pressures for adjustments or to external pressures for changes. Care providers who perceive having sufficient space report higher research use.** | |
| *Positive impact of context on implementation* | *Negative impact of context on implementation* |
|  | - Challenges to providing services across a geographical region [54][39] - Not all homes had a quiet space for staff to attend training [26] - Difficult to maintain resident privacy [44] |
| Domain: Organisational Slack-Time  **Definition: The cushion of actual or potential resources which allows an organization (unit) to adapt successfully to internal pressures for adjustments or to external pressures for changes. Care providers who perceive having sufficient time report higher research use.** | |
| *Positive impact of context on implementation* | *Negative impact of context on implementation* |
| - Staff not asked to deliver the intervention therefore achievable within working routines [55] (but implications for subsequent implementation) - Allowing time for dementia champions to develop new skills [8] | - Intervention was overambitious [37] - A longer or more intensive intervention may have fostered greater relationship-building with consequential clinical benefits [47][24] - Could not be accommodated within work load [11, 22, 34, 47] - Intervention needed to be flexible [41] - Required more time to achieve change [16, 24, 26, 30] - Took up too much staff time [3, 12, 22, 26][27] - Payment to cover extra hired staff, so that the RNs could be involved, but time involved in the data collection still unacceptable [13] |

1. Beer C, Horner B, Flicker L, Scherer S, Lautenschlager NT, Bretland N, et al. A cluster-randomised trial of staff education to improve the quality of life of people with dementia living in residential care: the DIRECT study. PLoS One. 2011;6:e28155.

2. Colon-emeric CS, Mcconnell E, Sandro O, Corazzini K, Porter K, Earp KM, et al. CONNECT for Better Fall Prevention in Nursing Homes: Results from a Pilot Intervention Study. J Am Geriatr Soc. 2014;61:2150–9.

3. Davison TE, Karantzas G, Mellor D, McCabe MP, Mrkic D. Staff-focused interventions to increase referrals for depression in aged care facilities: a cluster randomized controlled trial. Aging Ment Heal. 2013;17:449–55.

4. Ballard C, Orrell M, Sun Y, Moniz-Cook E, Stafford J, Whitaker R, et al. Impact of antipsychotic review and non-pharmacological intervention on health-related quality of life in people with dementia living in care homes: WHELD—a factorial cluster randomised controlled trial. Int J Geriatr Psychiatry. 2017;32:1094–103.

5. Husebo BS, Ballard C, Aarsland D, Selbaek G, Slettebo DD, Gulla C, et al. The Effect of a Multicomponent Intervention on Quality of Life in Residents of Nursing Homes: A Randomized Controlled Trial (COSMOS). J Am Med Dir Assoc. 2019;20:330–9.

6. Livingston G, Barber J, Marston L, Stringer A, Panca M, Hunter R, et al. Clinical and cost-effectiveness of the Managing Agitation and Raising Quality of Life (MARQUE) intervention for agitation in people with dementia in care homes: a single-blind, cluster-randomised controlled trial. The Lancet Psychiatry. 2019;6:293–304. doi:10.1016/S2215-0366(19)30045-8.

7. Palmer JA, Mor V, Volandes AE, McCreedy E, Loomer L, Carter P, et al. A dynamic application of PRECIS-2 to evaluate implementation in a pragmatic, cluster randomized clinical trial in two nursing home systems. Trials. 2018;19:453.

8. Fossey J, Garrod L, Tolbol Froiland C, Ballard C, Lawrence V, Testad I. What influences the sustainability of an effective psychosocial intervention for people with dementia living in care homes? A 9 to 12-month follow-up of the perceptions of staff in care homes involved in the WHELD randomised controlled trail. Int J Geriatr Psychiatry. 2019;34:674–82.

9. Rantz M, Zwygart-Stauffacher M, Hicks L, Mehr D, Flesner M, Petrovski GF, et al. Randomized Multilevel Intervention to Improve Outcomes of Residents in Nursing Homes in Need of Improvement. J Am Med Dir Assoc. 2012;4:60–8. doi:10.1016/j.jamda.2011.06.012.

10. De Visschere L, Schols J, van der Putten GJ, de Baat C, Vanobbergen J. Effect evaluation of a supervised versus non-supervised implementation of an oral health care guideline in nursing homes: a cluster randomised controlled clinical trial. Gerodontology. 2012;29:e96-106.

11. Anderson K, Bird M, Macpherson S, McDonough V, Davis T. Findings from a pilot investigation of the effectiveness of a snoezelen room in residential care: should we be engaging with our residents more? Geriatr Nurs. 2011;32:166–77.

12. Kinley, Stone L, Dewey M, Levy J, Stewart R, McCrone P, et al. The effect of using high facilitation when implementing the Gold Standards Framework in Care Homes programme: A cluster randomised controlled trial. Palliat Med. 2014;28:1099–109. doi:10.1177/0269216314539785.

13. Blekken LE, Nakrem S, Gjeilo KH, Norton C, Morkved S, Vinsnes AG. Feasibility, acceptability, and adherence of two educational programs for care staff concerning nursing home patients’ fecal incontinence: a pilot study preceding a cluster-randomized controlled trial. Implement Sci. 2015;10:72.

14. Brodaty H, Low LF, Liu Z, Fletcher J, Roast J, Goodenough B, et al. Successful ingredients in the SMILE study: Resident, staff, and management factors influence the effects of humor therapy in residential aged care. Am J Geriatr Psychiatry. 2014;22:1427–37. doi:10.1016/j.jagp.2013.08.005.

15. Stein-Parbury J, Chenoweth L, Jeon YH, Brodaty H, Haas M, Norman R. Implementing Person-Centered Care in Residential Dementia Care. Clin Gerontol. 2012;35:404-424 21p. doi:10.1080/07317115.2012.702654.

16. Chami K, Gavazzi G, Bar-Hen A, Carrat F, de Wazières B, Lejeune B, et al. A Short-Term, Multicomponent Infection Control Program in Nursing Homes: A Cluster Randomized Controlled Trial. J Am Med Dir Assoc. 2012;13:569.e9-569.e17 1p. doi:10.1016/j.jamda.2012.04.008.

17. Aasmul I, Husebo BS, Flo E. Description of an advance care planning intervention in nursing homes: outcomes of the process evaluation. BMC Geriatr. 2018;18:26.

18. Aasmul I, Husebo BS, Sampson EL, Flo E. Advance Care Planning in Nursing Homes - Improving the Communication Among Patient, Family, and Staff: Results From a Cluster Randomized Controlled Trial (COSMOS). Front Psychol. 2018;9:2284.

19. Boorsma M, Frijters DH, Knol DL, Ribbe ME, Nijpels G, van Hout HP. Effects of multidisciplinary integrated care on quality of care in residential care facilities for elderly people: a cluster randomized trial. Cmaj. 2011;183:E724-32.

20. Boyd M, Armstrong D, Parker J, Pilcher C, Zhou L, McKenzie-Green B, et al. Do gerontology nurse specialists make a difference in hospitalization of long-term care residents? Results of a randomized comparison trial. J Am Geriatr Soc. 2014;62:1962–7.

21. Wenborn J, Challis D, Head J, Miranda-Castillo C, Popham C, Thakur R, et al. Providing activity for people with dementia in care homes: a cluster randomised controlled trial. Int J Geriatr Psychiatry. 2013;28:1296–304.

22. Brooker DJ, Latham I, Evans SC, Jacobson N, Perry W, Bray J, et al. FITS into practice: translating research into practice in reducing the use of anti-psychotic medication for people with dementia living in care homes. Aging Ment Health. 2015;7863 March 2016:1–10. doi:10.1080/13607863.2015.1063102.

23. Luckett T, Chenoweth L, Phillips J, Brooks D, Cook J, Mitchell G, et al. A facilitated approach to family case conferencing for people with advanced dementia living in nursing homes: perceptions of palliative care planning coordinators and other health professionals in the IDEAL study. Int Psychogeriatrics. 2017;29:1713–22. doi:DOI: 10.1017/S1041610217000977.

24. Stern A, Mitsakakis N, Paulden M, Alibhai S, Wong J, Tomlinson G, et al. Pressure ulcer multidisciplinary teams via telemedicine: a pragmatic cluster randomized stepped wedge trial in long term care. BMC Heal Serv Res. 2014;14:83.

25. Close H, Hancock H, Mason JM, Murphy JJ, Fuat A, de Belder M, et al. “It’s Somebody else’s responsibility” - perceptions of general practitioners, heart failure nurses, care home staff, and residents towards heart failure diagnosis and management for older people in long-term care: a qualitative interview study. BMC Geriatr. 2013;13:69.

26. Ellard DR, Thorogood M, Underwood M, Seale C, Taylor SJ. Whole home exercise intervention for depression in older care home residents (the OPERA study): a process evaluation. BMC Med. 2014;12:1.

27. Surr CA, Holloway I, Walwyn REA, Griffiths AW, Meads D, Kelley R, et al. Dementia Care Mapping TM to reduce agitation in care home residents with dementia : The DCM ^TM^ EPIC cluster randomised controlled trial. Health Technol Assess (Rockv). 2019;:1–239.

28. Beer C, Lowry R, Horner B, Almeida OP, Scherer S, Lautenschlager NT, et al. Development and evaluation of an educational intervention for general practitioners and staff caring for people with dementia living in residential facilities. Int Psychogeriatr. 2011;23:221–9.

29. Simpson KM, Porter K, McConnell ES, Colon-Emeric C, Daily KA, Stalzer A, et al. Tool for evaluating research implementation challenges: a sense-making protocol for addressing implementation challenges in complex research settings. Implement Sci. 2013;8:2.

30. Agar M, Luckett T, Luscombe G, Phillips J, Beattie E, Pond D, et al. Effects of facilitated family case conferencing for advanced dementia: A cluster randomised clinical trial. PLoS One. 2017;12:1–16.

31. Colon-Emeric CS, McConnell E, Pinheiro SO, Corazzini K, Porter K, Earp KM, et al. CONNECT for better fall prevention in nursing homes: results from a pilot intervention study. J Am Geriatr Soc. 2013;61:2150–9.

32. Colon-Emeric CS, Pinheiro SO, Anderson RA, Porter K, McConnell E, Corazzini K, et al. Connecting the learners: improving uptake of a nursing home educational program by focusing on staff interactions. Gerontologist. 2014;54:446–59.

33. Dorsey ER, Deuel LM, Voss TS, Finnigan K, George BP, Eason S, et al. Increasing access to specialty care: a pilot, randomized controlled trial of telemedicine for Parkinson’s disease. Mov Disord. 2010;25:1652–9.

34. Beeckman D, Clays E, Hecke A, Vanderwee K, Schoonhoven L, Verhaeghe S. A multi-faceted tailored strategy to implement an electronic clinical decision support system for pressure ulcer prevention in nursing homes : A two-armed randomized controlled trial. Int J Nurs Stud. 2013;50:475–86. doi:10.1016/j.ijnurstu.2012.09.007.

35. Greenspan SL, Nace MD, Perera S FM et al. Lessons Learned from an Osteoporosis Clinical Trial in Frail Long Term Care Residents. Clin Trials. 2012;100:130–4.

36. Gage H, Dickinson A, Victor C, Williams P, Cheynel J, Davies SL, et al. Integrated working between residential care homes and primary care: a survey of care homes in England. BMC Geriatr. 2012;12:71.

37. Arendts G, Etherton-Beer C, Howard K, Lewin G, Sim M, Pickstock S, et al. Nurse led care coordination: trial protocol and development of a best practice resource guide for a cluster controlled clinical trial in Australian aged care facilities. Arch Gerontol Geriatr. 2014;58:15–9.

38. Kinley J, Stone L, Dewey M, Levy J, Stewart R, McCrone P, et al. The effect of using high facilitation when implementing the Gold Standards Framework in Care Homes programme: a cluster randomised controlled trial. Palliat Med. 2014;28:1099–109.

39. Meeks S, Van Haitsma K, Schoenbachler B, Looney SW. BE-ACTIV for depression in nursing homes: primary outcomes of a randomized clinical trial. J Gerontol B Psychol Sci Soc Sci. 2015;70:13–23.

40. Van Ness PH, Peduzzi PN, Quagliarello VJ. Efficacy and effectiveness as aspects of cluster randomized trials with nursing home residents: methodological insights from a pneumonia prevention trial. Contemp Clin Trials. 2012;33:1124–31.

41. Cohen-mansfield. What are the barriers to performing nonpharmalogical interventions for Behavioral symptoms in the nursing home? J Am Med Dir Assoc. 2012;100:130–4.

42. Shepherd V, Nuttall J, Hood K, Butler CC. Setting up a clinical trial in care homes: challenges encountered and recommendations for future research practice. BMC Res Notes. 2015;8:306.

43. Fossey J, Garrod L, Guzman A, Testad I. A qualitative analysis of trainer/coach experiences of changing care home practice in the Well-being and Health in Dementia randomised control trial. Dementia. 2018.

44. Hall S, Longhurst S, Higginson IJ. Challenges to conducting research with older people living in nursing homes. BMC Geriatr. 2009;9:38.

45. van der Kooij CH, Droes RM, de Lange J, Ettema TP, Cools HJ, van Tilburg W. The implementation of integrated emotion-oriented care: did it actually change the attitude, skills and time spent of trained caregivers? Dement. 2013;12:536–50.

46. Chi I, Law B V, Leung AC, Liu CP, Yeoh CS, Cheng YH, et al. Residential Assessment Instrument 2.0 in care planning for residents in nursing homes. Hong Kong Med J. 2010;16 Suppl 3:29–33.

47. Connolly MJ, Boyd M, Broad JB, Kerse N, Lumley T, Whitehead N, et al. The Aged Residential Care Healthcare Utilization Study (ARCHUS): A Multidisciplinary, Cluster Randomized Controlled Trial Designed to Reduce Acute Avoidable Hospitalizations From Long-Term Care Facilities. J Am Med Dir Assoc. 2015;16:49–55.

48. Fossey J, Garrod L, Lawrence V, Testad I, Stafford J, Murray J. “We should see her like part of the team”: an investigation into care home staff’s experiences of being part of an RCT of a complex psychosocial intervention. Aging Ment Heal. 2018;0:1–8. doi:10.1080/13607863.2018.1525603.

49. Crotty M, Halbert J, Rowett D, Giles L, Birks R, Williams H, et al. An outreach geriatric medication advisory service in residential aged care: a randomised controlled trial of case conferencing. Age Ageing. 2004;33:612-617 6p. http://search.ebscohost.com/login.aspx?direct=true&db=jlh&AN=106627237&site=ehost-live.

50. Simmons SF, Keeler E, An R, Liu X, Shotwell MS, Kuertz B, et al. Cost-Effectiveness of Nutrition Intervention in Long-Term Care. J Am Geriatr Soc. 2015;63:2308–16.

51. Vowden K, Vowden P. A pilot study on the potential of remote support to enhance wound care for nursing-home patients. J Wound Care. 2013;22:481–8.

52. Schnelle JF, Rahman A, Durkin DW, Beuscher L, Choi L, Simmons SF. A controlled trial of an intervention to increase resident choice in long term care. J Am Med Dir Assoc. 2013;14:345–51.

53. Dozeman E, Marwijk HWJ Van, Schaik DJF Van, Smit F, Stek ML, Horst E Van Der, et al. Contradictory effects for prevention of depression and anxiety in residents in homes for the elderly : a pragmatic randomized controlled trial. Int Pscychogeriatrics. 2012;24:1242–51.

54. Field TS, Tjia J, Mazor KM, Donovan JL, Kanaan AO, Harrold LR, et al. Randomized trial of a warfarin communication protocol for nursing homes: an SBAR-based approach. Am J Med. 2011;124:179.e1-7.

55. Greenspan S, Nace D, Perera S, Ferchak M, Fiorito G, Medich D, et al. Lessons learned from an osteoporosis clinical trial in frail long-term care residents. Clin Trials. 2012;9:247–56.
